# Documentation and Records: Harmonized GMP Requirements

**DOI:** 10.4103/0975-1483.80303

**Published:** 2011

**Authors:** KT Patel, NP Chotai

**Affiliations:** *Torrent Pharmaceuticals Ltd., R and D Center, Bhat, Ghandhinagar - 382 428, India*; 1 *AR College of Pharmacy and GH Patel Institute of Pharmacy, Vallabh-Vidhyanagar - 388 120, Gujarat, India*

**Keywords:** Documentation and records, good manufacturing practices, quality assurance

## Abstract

‘If it’s not written down, then it didn’t happen!’ The basic rules in any good manufacturing practice (GMP) regulations specify that the pharmaceutical manufacturer must maintain proper documentation and records. Documentation helps to build up a detailed picture of what a manufacturing function has done in the past and what it is doing now and, thus, it provides a basis for planning what it is going to do in the future. Regulatory inspectors, during their inspections of manufacturing sites, often spend much time examining a company’s documents and records. Effective documentation enhances the visibility of the quality assurance system. In light of above facts, we have made an attempt to harmonize different GMP requirements and prepare comprehensive GMP requirements related to ‘documentation and records,’ followed by a meticulous review of the most influential and frequently referred regulations.

## INTRODUCTION

### Tragic incident

It is a truism that it takes a disaster to happen for people, and especially regulators, to wake up and review the accepted way of doing things. So, too, with the issue of drug safety and drug quality.[1]

The 1972 Devonport, UK, incident resulted in at least five deaths when drug products designed to be sterile became contaminated and recipients developed infections. An unwritten change to autoclave operation, communicated orally between operators, resulted in dextrose intravenous solutions that were not uniformly sterile. The Clothier inquiry, which examined the causes and contributing factors, identified several violations of what we now consider basic good manufacturing practice (GMP).

The chain of events that compromised the safety of the drug product included inadequate maintenance, inadequate understanding of autoclave operation, and regular deviations from the written production instructions (often as an attempt to compensate for equipment malfunction). Together, these factors resulted in a sterilization cycle that did not assure that all vials in the autoclave were sterilized; thus, some doses were safe, while others led to sepsis in patients who received them. This incident helped to define sterility assurance in an operational way. Processes and requirements for equipment validation were created, and legal right of inspection was explicitly given to the agency.

Validation was developed as a means of documenting systematic evaluation of the sterilization cycle — building in a safety factor — and identifying the critical parameters that need to be controlled to assure process performance. The concept that quality must be designed into the process and cannot be achieved only by testing remains a central tenet of current good manufacturing practice (cGMP). In other words, how you make something helps to define its level of quality. Preventing errors is more effective than finding rejects because it is not possible to detect all rejects.[2] The current requirement for ’documented evidence’ may be driven by this event of Devenport.

## GOOD MANUFACTURING PRACTICES

GMP is that part of quality assurance which ensures that products are consistently produced and controlled to the quality standards appropriate to their intended use. GMP is aimed primarily at diminishing the risk inherent in any pharmaceutical production. Such risks are essentially of two types: cross-contamination (in particular, with unexpected contaminants) and mix-ups (for example, false labeling).[3]

Worldwide, there are different official regulatory statements and guidelines, both national and international, for GMP for pharmaceutical (or ‘drug’ or ‘medicinal’) products. They may be regulations (as in the US, Japan, or Korea), directives (as in the EU), guides (as in the UK), codes (as in Australia), or a WHO code (as in many Southeast Asia Countries). Among them, the following stand out as the most influential and most frequently referenced:

- The US Current Good Manufacturing Practices for Finished Pharmaceuticals regulations (the US cGMPs).[4]
- The Guide to Good Manufacturing Practice for Medicinal Products of the European Union (the EC GMP Guide).[5]
- The ICH Q7 Good Manufacturing Practice Guide for Active Pharmaceutical Ingredients.[6]
- The World Health Organization (WHO) good manufacturing practices.[7]

The other guidelines and regulations referred by the pharmaceutical manufacturers are as under:

- Schedule M ‘Good Manufacturing Practices and Requirements of Premises, Plant and Equipment for Pharmaceutical Products,’ The Drugs and Cosmetics Act and Rules, India.[8]
- PIC/S Guide to Good Manufacturing Practice for Medicinal Products.[9]
- Center for Drug Evaluation and Research (CDER): Manufacturing, Processing, or Holding Active Pharmaceutical Ingredients.[10]

## DOCUMENTATION

Documentation is the key to GMP compliance and ensures traceability of all development, manufacturing, and testing activities. Documentation provides the route for auditors to assess the overall quality of operations within a company and the final product.

### The 10 golden rules of GMP[11]

Table 1 describes the 10 golden rules of GMP. Rule No. 3 and 5 describe the importance of documentation and records.

**Table 1:** The 10 golden rules of GMP

Number	The golden rule
1	Get the facility design right from the start
2	Validate processes
3	Write good procedures and follow them
4	Identify who does what
5	Keep good records
6	Train and develop staff
7	Practice good hygiene
8	Maintain facilities and equipment
9	Build quality into the whole product lifecycle
10	Perform regular audits

### Basics[12]

- The management of each operational site is required to define responsibility for origination, distribution, maintenance, change control, and archiving of all GMP documentation and records within that department or unit.
- Document owners are required to ensure that all aspects of documentation and records management specified in form of standard operating procedures (SOPs).
- All associates have the responsibility of ensuring that all GMP activities are performed according to the official SOPs; any deviations in procedure are reported to their supervisor and are adequately documented.
- The local quality assurance unit has the responsibility of ensuring via organizational measures and auditing that GMP documentation and records systems used within the operational unit are complete and comply with the relevant GMP requirements, and also that the requirements of the SOPs are followed.
- Requirements for specific documents or record, including ownership, content, authorization, and change control procedures, has to be described or cross-referenced in the quality modules which relate to the subject of the document.

### General requirements[13]

- Good documentation constitutes an essential part of the quality assurance system. Clearly written procedures prevent errors resulting from spoken communication, and clear documentation permits tracing of activities performed.
- Documents must be designed, prepared, reviewed, and distributed with care.
- Documents must be approved, signed, and dated by the appropriate competent and authorized persons.
- Documents must have unambiguous contents. The title, nature, and purpose should be clearly stated. They must be laid out in an orderly fashion and be easy to check. Reproduced documents must be clear and legible.
- Documents must be regularly reviewed and kept up-to-date. When a document has been revised, systems must be operated to prevent inadvertent use of superseded documents (e.g., only current documentation should be available for use).
- Documents must not be handwritten; however, where documents require the entry of data, these entries may be made in clear legible handwriting using a suitable indelible medium (i.e., not a pencil). Sufficient space must be provided for such entries.
- Any correction made to a document or record must be signed or initialed and dated; the correction must permit the reading of the original information. Where appropriate, the reason for the correction must be recorded.
- Record must be kept at the time each action is taken and in such a way that all activities concerning the conduct of preclinical studies, clinical trials, and the manufacture and control of products are traceable.
- Storage of critical records must at secure place, with access limited to authorized persons. The storage location must ensure adequate protection from loss, destruction, or falsification, and from damage due to fire, water, etc.
- Records which are critical to regulatory compliance or to support essential business activities must be duplicated on paper, microfilm, or electronically, and stored in a separate, secure location in a separate building from the originals.
- Date may be recorded by electromagnetic or photographic means, but detailed procedures relating to whatever system is adopted must be available. Accuracy of the record should be checked as per the defined procedure. If documentation is handled by electronic data processing methods, only authorized persons should be able to enter or modify data in the computer, access must be restricted by passwords or other means, and entry of critical data must be independently checked.
- It is particularly important that during the period of retention, the data can be rendered legible within an appropriate period of time.
- If data is modified, it must be traceable.

There are various types of procedures that a GMP facility can follow. Given below is a list of the most common types of documents, along with a brief description of each.

1. *Quality manual*: A global company document that describes, in paragraph form, the regulations and/or parts of the regulations that the company is required to follow.
2. *Policies*: Documents that describe in general terms, and not with step-by-step instructions, how specific GMP aspects (such as security, documentation, health, and responsibilities) will be implemented.
3. *Standard operating procedures (SOPs)*: Step-by-step instructions for performing operational tasks or activities.
4. *Batch records*: These documents are typically used and completed by the manufacturing department. Batch records provide step-by-step instructions for production-related tasks and activities, besides including areas on the batch record itself for documenting such tasks.
5. *Test methods*: These documents are typically used and completed by the quality control (QC) department. Test methods provide step-by-step instructions for testing supplies, materials, products, and other production-related tasks and activities, e.g., environmental monitoring of the GMP facility.
  Test methods typically contain forms that have to be filled in at the end of the procedure; this is for documenting the testing and the results of the testing.
6. *Specifications*: Documents that list the requirements that a supply, material, or product must meet before being released for use or sale. The QC department will compare their test results to specifications to determine if they pass the test.
7. *Logbooks*: Bound collection of forms used to document activities. Typically, logbooks are used for documenting the operation, maintenance, and calibration of a piece of equipment. Logbooks are also used to record critical activities, e.g., monitoring of clean rooms, solution preparation, recording of deviation, change controls and its corrective action assignment.

### Hierarchical document system[12]

**Figure 1:**
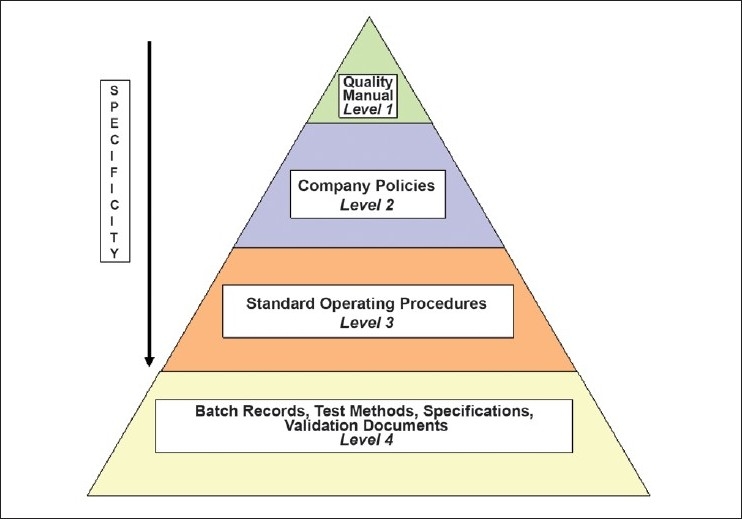
Hierarchical document system

- The organization should establish a hierarchical document system as mentioned in Figure 1:
- The regulations that a company is responsible for following (e.g., USFDA/EU GMP/ICH/Schedule M, etc.) should be at the top of the document pyramid and should govern the directives of the sublevels.
- The level immediately beneath the regulations, level 1 documents (e.g., the Quality Manual), should break the regulations into parts specific to those that the company is required to follow. These documents should establish overall principles and guidelines for how the company plans on developing, documenting, and implementing a cCMP-compliant quality system. Top-level documents apply to all departments within a cGMP-compliant company and are not specific in nature.
- The next level, level 2, of documents in the hierarchical document pyramid should further break down the parts of the regulations into specific subjects or topics. These documents (e.g., Company Polices) should establish guidelines with which all subordinate level procedures must comply to ensure consistency across departments.
- Level 2 documents should not provide specific directive instructions or forms for documenting data but rather provide the overall intentions and guidelines governing critical programs or systems as well as explanation for the rationale and program designs. These documents will apply to all departments within a GMP-compliant company.
- SOPs should be the next level in the document hierarchy after company policy documents. These types of documents should provide specific step-by-step instructions for performing the operational tasks or activities that were talked about in the previous levels (for example: SOP titled ’Writing, Revising, Numbering, and Distributing Controlled Documents’). Level 3 documents (i.e., SOPs) should be department specific or function specific.
- The last level of documents in a document hierarchical structure are level 4 documents. These documents are the most specific in nature, (e.g., batch record, test methods, validation procedures). They apply to a specific department, product, equipment, or process. Level 4 documents provide step-by-step instructions for production-related tasks and activities as well as provide a means for documenting such tasks using, for example, data sheets, forms, or batch records. The details outlined in these documents may override directions given in other level documents. (For example: the company’s documentation SOP may state that numbers be rounded off to three significant figures; the batch record, on the other hand, may state that all numbers be expressed in scientific notation. Thus, instructions in level 4 documents, which are specific to a particular process, can overrule the instruction mentioned in level 3 documents, which are general in nature. The document hierarchy pyramid is one way of organizing a company’s documents.

More/less levels may be added/subtracted to meet the company’s specific needs.

## HARMONIZED REQUIREMENT

The harmonized requirements were prepared after taking into consideration the above mentioned guidance documents/regulatory requirements.[4–10]

### Site master file

The manufacturer should prepare a succinct document in the form of a ‘Site Master File,’ containing specific and factual GMP about the production and/or control of pharmaceutical manufacturing procedures carried out at the premises. It should contain the descriptions of the following:

#### General information:

- Brief information on the firm
- Pharmaceutical manufacturing activities, as permitted by the licensing authority
- Other manufacturing activities, if any, carried out on the premises
- Type of products licensed for manufacture, with flowcharts detailing procedure and process flow
- Number of employees engaged in the production, quality control, storage and distribution
- Use of outside scientific, analytical, or other technical assistance in relation to manufacture and analysis
- Short description of the quality management system of the firm
- Products details registered with foreign countries

#### Personnel:

- Organizational chart showing the arrangements for quality assurance, including production and quality control
- Qualification, experience, and responsibilities of key personnel

#### Premises:

- Simple plan or description of manufacturing areas drawn to scale
- Nature of construction and fixtures/fittings
- Brief description of ventilation systems. More details should be given for critical areas with potential risk of airborne contamination (schematic drawing of systems). Classification of the rooms used for the manufacture of sterile products should be mentioned.
- Special areas for the handling of highly toxic, hazardous, and sensitizing materials.
- Brief description of the water system (schematic drawings of systems), including sanitation.
- Description of planned preventive maintenance programs for premises and of the recording system.

#### Equipment:

- Brief description of major equipment used in production and in the quality control laboratories (a list of equipment required)
- Description of planned preventive maintenance programs for equipment and of the recording system
- Qualification and calibration, including the recording systems, and arrangements for computerized systems validation

#### Sanitation:

- Written specifications and procedures for cleaning manufacturing areas and equipment

#### Documentation:

- Arrangements for the preparation, revision, and distribution of documents
- Necessary documentation for the manufacture
- Any other documentation related to product quality that is not mentioned elsewhere (e.g., regarding microbiological control of air and water)

#### Production:

- Brief description of production operations using, wherever possible, flow sheets and charts specifying important parameters
- Arrangements for the handling of starting materials, packaging materials, and bulk and finished products; this includes the arrangements for sampling, quarantine, release, and storage.
- Arrangements for the handling of rejected materials and products.
- Brief description of the general policy for process validation.

#### Quality control:

- Description of the quality control system and of the activities of the quality control department. Procedures for the release of the finished products.

#### Loan license manufacture and licensee:

- Description of the way in which compliance with GMP by the loan licensee should be assessed.

#### Distribution, complaints, and product recall:

- Arrangements and recording system for distribution
- Arrangements for the handling of complaints and product recalls

#### Self inspection:

- Short description of the self-inspection system, indicating whether an independent and experienced external expert is to be involved in evaluating the manufacturer’s compliance with GMP in all aspects of production

#### Export of drugs

- Products exported to different countries
- Complaints and product recall, if any

### Documentation system and specifications

Documentation is an essential part of the quality assurance system and, as such, should be related to all aspects of GMP. Its aim is to define the specifications for all materials and the method of manufacture and control, to ensure that all personnel concerned with manufacture have the information necessary to decide whether or not to release a batch of a drug for sale, and to provide an audit trail that will permit investigation of the history of any suspected defective batch. The specifications should describe in detail the requirements with which the products or materials used or obtained during manufacture have to conform. They serve as a basis for quality evaluation.

Manufacturing formulae and processing and packaging instructions should specify all the starting materials used and describe all processing and packaging operations. Procedures should give directions for performing certain operations, e.g., cleaning, clothing, environmental control, sampling, testing, and equipment operation. Records should provide a history of each batch of product, including its distribution, and also of all other relevant circumstances pertinent to the quality of the final product.

Written records should be maintained so that data can be used for evaluating, at least annually, the quality standards of each drug product to determine the need for changes in drug product specifications or manufacturing or control procedures. Written procedures should be established and followed for such evaluations and must include provisions for:

- A review of a representative number of batches, whether approved or rejected and, where applicable, the records associated with the batch.
- A review of complaints, recalls, and returned or salvaged drug products, and of the investigations conducted.

All documents related to the manufacture of intermediates, active pharmaceutical ingredients (API), and finished products should be prepared, reviewed, approved, and distributed according to written procedures. Such documents can be paper-based or in electronic form. Documents should be approved, signed, and dated by the appropriate responsible persons. No document should be changed without authorization and approval.

Each specification for raw materials, intermediates, final products, and packing materials should be approved and maintained by the quality control department. Periodic revisions of the specifications must be carried out whenever changes are necessary.

The issuance, revision, superseding, and withdrawal of all documents should be controlled, with maintenance of revision histories. When a document has been revised, systems should be operated to prevent inadvertent use of superseded documents. Superseded documents should be retained for a specific period of time.

Periodic revisions of the specifications may be necessary to comply with new editions of the national pharmacopoeia or other official compendia.

Documents should have unambiguous contents: the title, nature, and purpose should be clearly stated. They should be laid out in an orderly fashion and be easy to check. Reproduced documents should be clear and legible. The process of reproduction of working documents from master documents must not allow any error to be introduced through the reproduction process.

A procedure should be established for retaining all appropriate documents (e.g., development history reports, scale-up reports, technical transfer reports, process validation reports, training records, production records, control records, and distribution records). The retention periods for these documents should be specified.

All production, control, and distribution records should be retained for at least 1 year after the expiry date of the batch. For APIs with retest dates, records should be retained for at least 3 years after the batch is completely distributed.

Documents should not be handwritten; however, where documents require the entry of data, these entries may be made in clear, legible, indelible handwriting. Sufficient space should be provided for such entries. Any alteration made to the entry on a document should be signed and dated; the alteration should permit the reading of the original information. Where appropriate, the reason for the alteration should be recorded.

During the retention period, originals or copies of records should be readily available at the establishment where the activities described in such records occurred. Records that can be promptly retrieved from another location by electronic or other means are acceptable.

Data may be recorded by electronic data processing systems or photographic or other reliable means, but detailed procedures relating to the system in use should be available and the accuracy of the records should be checked. If documentation is handled by electronic data processing methods, only authorized persons should be able to enter or modify data in the computer, and there should be a record of changes and deletions. Access should be restricted by passwords or other means and the result of entry of critical data should be independently checked. Batch records that are electronically stored should be protected by back-up transfer onto magnetic tape, microfilm, paper, or other means.

Specifications should be established and documented for raw materials, intermediates (where necessary), and API/formulations, as well as for labeling and packaging materials. In addition, specifications may be appropriate for certain other materials, such as process aids, gaskets, or other materials used during the production of intermediates or API/formulations that could critically impact on quality. Acceptance criteria should be established and documented for in-process controls.

If electronic signatures are used on documents, they should be authenticated and secure.

### Equipment cleaning and use record

Records of major equipment use, cleaning, sanitization and/or sterilization, and maintenance should show the date, time (if appropriate), product, and batch number of each batch processed in the equipment and the name and signature of the person who has performed the cleaning and maintenance. The persons performing and double-checking the cleaning and maintenance should date and sign or initial the log, indicating that the work was performed. Entries in the log should be in chronological order.

Cross-contamination should be avoided by appropriate technical or organizational measures, for example:

- Production in segregated areas (required for products such as the penicillins, live vaccines, live bacterial preparations, and some other biologicals), or by campaign (separation in time) followed by appropriate cleaning
- Providing appropriate air-locks and air extraction
- Minimizing the risk of contamination caused by recirculation or re-entry of untreated or insufficiently treated air
- Keeping protective clothing inside areas where products with special risk of cross-contamination are processed
- Using cleaning and decontamination procedures of known effectiveness, as ineffective cleaning of equipment is a common source of cross-contamination
- Using ‘closed systems’ of production
- Testing for residues and use of cleaning status labels on equipment

If equipment is dedicated to manufacturing one intermediate or API, then individual equipment records of different activities like cleaning, maintenance, batch log, etc., are not necessary, provided the batch record has complete traceability of this information. In case of formulation manufacturing, the appropriate cleaning procedure should be established to ensure removal of any residue of the previous product.

### Records of raw materials, intermediates, labeling, and packaging materials

Records should be maintained, including:

- The name of the manufacturer; identity and quantity of each shipment of each batch of raw materials, intermediates, or labeling and packaging materials; the name of the supplier; the supplier’s control number(s) (if known) or other identification number; the number allocated on receipt; and the date of receipt;
- The results of any test or examination performed and the conclusions derived from this;
- Records tracing the use of materials;
- Documentation of the examination and review of labeling and packaging materials for conformity with established specifications;
- The final decision regarding rejected raw materials, intermediates, or labeling and packaging materials.

Starting materials in the storage area should be appropriately labeled. Labels should bear at least the following information:

- The designated name of the product and the internal code reference, where applicable
- The batch number given by the supplier and, on receipt, the control or batch number (if any) given by the manufacturer; these must be documented so as to ensure traceability
- The status of the contents (e.g., on quarantine, on test, released, rejected, returned, recalled, etc.)
- Where appropriate, an expiry date or a date beyond which retesting is necessary

Master (approved) labels should be maintained for comparison with issued labels.

### Master production instructions/master production and control records (MPCR)/master formula card (MFC)

To ensure uniformity from batch to batch, master production instructions for each intermediate or API/finished product should be prepared, dated, and signed by one person and independently checked, dated, and signed by a second person in the quality unit(s).

Competent persons experienced in production and quality control should be responsible for the content and distribution within the firm of instructions and master formulae. These should be duly signed and dated.

Outdated master formulae should be withdrawn but retained for reference. Copies of the master formula should be prepared in a manner that will eliminate any possibility of transcription error.

In certain circumstances, for example, in the first production runs following pilot development, the master formula might need to be amended. Any amendments must be formally authorized and signed by competent person(s). The amended document should be replaced at the earliest opportunity by a newly prepared master formula.

Processing should be carried out in accordance with the master formula. Master production instructions should include:

- The name of the intermediate/API/formulation being manufactured and an identifying document reference code, if applicable
- A complete list of raw materials and intermediates (designated by names or codes sufficiently specific to identify any special quality characteristics)
- An accurate statement of the quantity or ratio of each raw material or intermediate to be used, including the unit of measure. Where the quantity is not fixed, the calculation for each batch size or rate of production should be included. Variations to quantities should be included wherever justified
- The production location and major production equipment to be used
- Detailed production instructions, including the:
  □ Sequences to be followed
  □ Ranges of process parameters to be used
  □ The methods, or reference to the methods, to be used for preparing the critical equipment (e.g., cleaning, assembling)
  □ Sampling instructions and in-process controls, with their acceptance criteria, where appropriate
  □ Time limits for completion of individual processing steps and/or the total process, where appropriate
  □ Expected yield ranges at appropriate phases of processing or time.
- Where appropriate, special notations and precautions to be followed, or cross-references to these
- Instructions for storage of the intermediate or API/semi-finished formulations to assure its suitability for use; instructions should cover the labeling (specimen labels and packaging materials and special storage conditions with time limits, where appropriate).

### Batch production records/batch production and control records (BPCR)/batch manufacturing record (BMR)

Batch production records should be prepared for each intermediate and API/formulation and should include complete information relating to the production and control of each batch. The batch production record should be checked before issuance to assure that it is the correct version and a legible accurate reproduction of the appropriate master production instruction. If the batch production record is produced from a separate part of the master document, that document should include a reference to the current master production instruction being used.

Before any processing begins, a check should be performed and recorded to ensure that the equipment and workstation are clear of previous products, documents, or materials not required for the planned process and that the equipment is clean and suitable for use.

These records should be numbered with a unique batch or identification number and dated and signed when issued. In continuous production, the product code together with the date and time can serve as the unique identifier until the final number is allocated.

The batch number should be immediately recorded in a logbook or by electronic data processing system. The record should include date of allocation, product identity, and size of batch.

Documentation of completion of each significant step in the batch production records (batch production and control records) should include:

- Dates and, when appropriate, times
- Identity of major equipment used (e.g., reactors, driers, mills, etc.)
- Specific identification of each batch, including weights, measures, and batch numbers of raw materials, intermediates, or any reprocessed materials used during manufacturing
- Actual results recorded for critical process parameters
- Any sampling performed
- Signatures of the persons performing and directly supervising or checking each critical step in the operation
- In-process and laboratory test results
- Actual yield at appropriate phases or times
- Description of packaging and label
- Representative label (commercial supply)
- Any deviation noted, its evaluation, and investigation conducted (if appropriate) or reference to that investigation (if stored separately)
- Results of release testing
- All analytical records relating to the batch, or a reference that will permit their retrieval
- A decision for the release or rejection of the batch, with the date and signature of the person responsible for the decision
- The production record review

Production and quality control records should be reviewed as part of the approval process of batch release. Any divergence or failure of a batch to meet its specifications should be thoroughly investigated. The investigation should, if necessary, extend to other batches of the same product and other products that may have been associated with the specific failure or discrepancy. A written record of the investigation should be made and should include the conclusion and follow-up action.

The following information should be recorded at the time each action is taken (the date must be noted and the person responsible should be clearly identified by signature or electronic password):

- The name of the product, the batch number and the quantity of product to be packed, as well as the quantity actually obtained and its reconciliation
- The date(s) and time(s) of the packaging operations
- The name of the responsible person carrying out the packaging operation
- The initials of the operators of the different significant steps
- The checks made for identity and conformity with the packaging instructions, including the results of in-process controls
- Details of the packaging operations carried out, including references to equipment and the packaging lines used and, when necessary, instructions for keeping the product unpacked or a record of returning product that has not been packaged to the storage area
- Whenever possible, the regular check for correctness of printing (e.g. batch number, expiry date and other additional overprinting) and specimen samples collected
- Notes on any special problems, including details of any deviation from the packaging instructions, with written authorization by an appropriate person
- The quantities and reference number or identification of all printed packaging materials and bulk product issued, used, destroyed, or returned to stock and the quantities of product obtained; this is necessary to permit an adequate reconciliation.

### Laboratory control records

Laboratory control records should include complete data derived from all tests conducted to ensure compliance with established specifications and standards, including examinations and assays, as follows:

- A description of samples received for testing, including the material name or source, batch number and, where appropriate, the manufacturer and/or supplier; alternatively, other distinctive code, date of sample taken and, where appropriate, the quantity of the sample and date the sample was received for testing
- A statement of, or reference to, each test method used
- A statement of the weight or measure of sample used for each test as described by the method; data on, or cross-reference to, the preparation and testing of reference standards, reagents, and standard solutions
- A complete record of all raw data generated during each test, in addition to graphs, charts, and spectra from laboratory instrumentation, all properly identified to show the specific material and the batch tested
- A record of all calculations performed in connection with the test including, for example, units of measure, conversion factors, and equivalency factors
- A statement of the test results and how they compare with established acceptance criteria
- The signature of the person who performed each test and the date(s) on which the tests were performed
- The date and signature of a second person, showing that the original records were reviewed for accuracy, completeness, and compliance with established standards.

Complete records should also be maintained for:

- Any modifications to an established analytical method
- Periodic calibration of laboratory instruments, apparatus, gauges, and recording devices
- All stability testing performed on APIs/formulations
- Out-of-specification (OOS) investigations

Complete records should be maintained of any testing and standardization of laboratory reference standards, reagents, and standard solutions; record should also be maintained of periodic calibration of laboratory instruments, apparatus, gauges, and recording devices.

### Batch production record review

Written procedures should be established and followed for the review and approval of batch production and laboratory control records, including packaging and labeling, to determine compliance of the intermediate or API with established specifications before a batch is released or distributed.

Batch production and laboratory control records of critical process steps should be reviewed and approved by the quality unit(s) before an API batch is released or distributed. Production and laboratory control records of non-critical process steps can be reviewed by qualified production personnel or other units, following procedures approved by the quality unit(s).

All deviation, investigation, and OOS reports should be reviewed as part of the batch record review before the batch is released.

The quality unit(s) can delegate to the production unit the responsibility and authority for release of intermediates, except for those shipped outside the control of the manufacturing company.

Distribution record should be maintained and must include the batch number; quantity produced; name, address, and contact details of customer; quantity supplied; and date of supply.

## POLICY FOR IMPLEMENTATION

The following approach pertaining to ‘documentation and records’ may be helpful for pharmaceutical manufacturers to meet the expectations of different regulatory agencies.

### Write good procedures and follow them[11]

Think about what happens in a workplace if written procedures are not available. People rely on more senior employees to tell them how to do things and then do their job from memory. This is fine for a company making garden pots, but not so good when the products being made are pharmaceuticals and can even cause death!

In the food, drug, and medical device industry it is critical that good procedures are in place to ensure a controlled and consistent performance; it is an essential part of GMP. Procedures should be clear, concise, and logical. Consider hiring a professional technical writer to do the job. Unlike permanent employees, they know how write well and will perform usability tests to ensure that the documents work. Review of procedure by an independent party can also help to improve process.

Outline the task before you begin writing the procedure. Create a brief breakdown of the important steps and key points related to the task; a flowchart is a useful tool. Remember that people do not usually read procedures from start to finish; they tend to scan the document for key words. To make information easier to digest and follow, break the procedure into chunks and use the following:

- Headings
- Tables
- Bullet points
- Diagrams

When writing out any procedure, one should try and visualize the person who will be following that procedure. Use language that that person can understand. Do not include too much or too little information. Increase the readability of the instructions by using simple sentences and by writing in a conversational style. Most companies have a 3-year review cycle for their documents; however, this can be set according to the likelihood of change in the process that the document relates to.

### Following procedures[11]

It is all very well to have great written procedures in place but to ensure a controlled and consistent performance they need to be followed; it is a GMP requirement. Frequently, the steps described in a written procedure may not appear to be the most efficient way of working. Taking shortcuts may save time or make the task easier, but one should never deviate from a written procedure without the approval of a supervisor or the quality department.

There are two main reasons for this:

- Many shortcuts may create pitfalls that can be costly in the end.
- Each step in a procedure has been included for a purpose.

Even though the rationale of a particular step may not be immediately apparent, it may have been put there as a check for another stage of the process. Ideas for improvement should always be encouraged, but do not change procedures without assessing the impact on the entire process.

### Keep good records[11]

Good records enable one to track all activities performed during batch manufacture, from the receipt of raw materials to the final product release; they provide a history of the batch and its distribution. It is an essential part of GMP to keep accurate records, and during an audit it helps convey the message that procedures are being followed. It also demonstrates that the processes are known and are under control.

### Remember!!!

- Record all necessary information immediately upon completion of a task
- Never trust your memory or write results on loose pieces of paper
- Write your name legibly in ink. Remember that by signing records you are certifying that the record is correct and that you have performed the task as per the defined procedure.
- Draw a single line through any mistakes, and initial and date the correction. Include a reason for the correction at the bottom of the page.
- Record details if you deviate from a procedure. Ask your supervisor or the quality department for advice if a deviation should occur.
- Do not document someone else’s work unless you are designated and trained to do so.
- Never assume that undocumented work has been properly completed – if it’s not written down, then it didn’t happen!

### Documents/SOPs required

The following documents and procedures should be prepared to fulfill the above mentioned requirements. The data generated through these procedures should be maintained to show compliance with the above mentioned requirements.

- Prepare apex documents like Quality Policy, Quality Manual, Site Master File, Validation Master Plan, etc. to describe the quality commitments of the management
- Define the roles and responsibilities of all personnel working in the organization
- Prepare policy for periodic review of documents. Ensure that the current industrial practices and pharmacopoeial requirements are fulfilled by the current versions of documents
- SOP for document (SOPs, MPCR, BPCR, validation/qualification protocols, formats) preparation, review, approval, training, distribution, control, and its retention
- Procedure for maintaining revision history
- Management, control, and retention of superseded or obsolete documents
- Document archival and retrieval procedure
- Handling, archival, retrieval, and retention of electronic records/documents
- Procedure for control of electronic signatures
- Equipment cleaning and sanitation procedure
- Issuance and control of equipment logs
- Document describing measures taken for avoidance of cross-contamination and its training records
- Cleaning validation master plan
- Procedure for batch-to-batch and product-to-product cleaning and its verification to ensure removal of residue of previous batch/product
- Records for incoming raw materials and packaging materials
- SOP for preparation of process validation protocol and reports
- SOP for preparation of master production control records
- SOP for preparation of batch manufacturing and control records
- SOP for allocation of batch number
- Calibration master plan and calibration reports
- Batch release procedure
- SOP for preparation and control of QC data sheet
- SOP for allocation of analytical control number
- Procedure for review of analytical data
- SOP for investigation of OOS results
- SOP for change control, revision of any process or documents, or upgradation of facility or equipment should be routed through impact assessment and change control procedure
- SOP for deviation handling system
- SOP for corrective and preventive action (CAPA)
- SOP for stability testing
- SOP for product distribution and its control

## CHECKLIST FOR COMPLIANCE ASSESSMENT

The following checkpoints/checklist may help to assess the compliance of ‘documentation and records’ with GMP requirements

## CONCLUSION

Pharmaceutical manufacture and regulation is clearly an international business. With the increasing emphasis on harmonization efforts and standard setting, as well as mutual recognition agreements, knowledge of foreign regulations is a must both for understanding the future direction of these efforts as well as for international supply of drug products. It is anticipated that the approach described here will be a useful reference work for those personnel preparing and using documents for pharmaceutical manufacture. It can serve as a tool for training staff and may prove to be useful for quality assurance professionals for assessment of compliance during self-inspection. It is again emphasized that documentation is a very important aspect of GMP and will enhance the visibility of the quality assurance function.

**Table:** 

Checklist/checkpoints		Document to refer	Y/N/NA	Remarks
•	Is there an SOP for writing, handling, and updating SOPs?	SOP for SOP		
•	Are all documents passing through appropriate review and approval procedure?	Template of all document		
•	Distribution records maintained for all documents?	Traceability of any specification/SOP		
•	Is there any procedure for ensuring that the current version of the documents are being used?	Document distribution and retrieval record		
•	Is responsibility assigned for issuance and control of documents?	Job description		
•	Is history of changes made in documents maintained?	Document history record		
•	Does document control procedure include the procedure for handling obsolete versions?	Traceability of compliance		
•	Does the storage/archival of documents provide a suitable environment to minimize deterioration or damage to quality-related documents?	Archive		
•	Is there a system for periodic review of documents?	Document review/revision SOP		
•	Is there a document control system available?	Respective SOP		
•	Are all quality-related documents being retained for history?	Superseded/obsolete document		
•	Are corrections made in documents signed and explained? Does the SOP reflect this policy?	SOP for correction of entries		
•	Are electronic signatures used? If yes, is there an adequate control or security measure?	SOP		
•	Is equipment cleaning being recorded in the logbook?	Logbook		
•	Is preventive maintenance activity being recorded in the logbook? Or is there any other appropriate documentation?	Preventive maintenance plan and logbook		
•	Is RM available at the warehouse, labeled with the following details:	In RM store		
	- Lot No.			
	- Receipt date			
	- Approval/status label			
•	Are master labels retained?	BPR / BPCR		
•	(For all lots that are packed and supplied, master labels should be part of Batch Packaging Record (BPR) or be separately filed)			
•	Does the MPCR/MFC mention the following details?	MPCR/MFC		
	-Name of material, with code			
	-Quantity			
	-Rationale			
	-Equipment to be used			
	-Process parameter			
	-In-process checks			
	-Sampling instruction			
	-Expected yield			
•	Have process parameters critical to quality been defined and, if parameters are exceeded, is the affect on quality known?	MPCR and development report		
•	Is there a system for identifying major equipment, instruments, and production lines? Is this information included in batch production and control records where appropriate?	MPCR and BPCR		
•	Is there a system to determine customer requirements related to the product and supply of the product?	Policy and evident document		
•	Is there a formal procedure to communicate the agreed upon customer requirements to the appropriate personnel?	SOP and evident document		
•	Is there a procedure in place to assure that the manufacturer and the customer have mutually agreed upon the specifications and other requirements? If not, what is the alternative process?	Agreement		
•	Is there a system to assure that any mutually agreed customer-initiated changes are promptly incorporated?	Agreement		
•	Is there an adequate system in place to assure that significant process changes, including the use of subcontractors and their effect on the product, are communicated to the customer?	Agreement		
•	Does the batch record mention the following:	BPCR		
	-Deviations, if any			
	-In-process results			
	-Release statement			
	-All details should match with MPCR			
•	Are specifications for all material available with QC and user department?	Traceability at QC		
•	Is method of analysis available with QC?	Traceability at QC		
•	Is there an SOP for investigation of OOS?	SOP and evident document		
•	Does the analytical report/COA mention the reference of STP used?	Analytical report		
•	Is analysis being performed by qualified personnel?	Analyst and its qualification and copy of FDA approval		
•	Are batch record and analytical records being reviewed by QA before dispatch?	SOP on batch release

GMP: Good manufacturing practice, cGMP: Current good manufacturing practice, SOP: Standard operating procedures, QC: Quality control, MFC: Master formula card, MPCR: Master production and control records, BMR: Batch manufacturing record, BPR: Batch packaging record, BPCR: Batch production and control records, API: Active pharmaceutical ingredients, OOS: Out-of-specification, RM: Raw material, COA: Certificate of analysis, STP: Standard test procedure, QA: Quality assurance

## References

[1] Website of Globepharm. The Advent of GMPs. http://www.globepharm.org/what-is-gmp/international-GMPs/advent-of-gmps.html.

[2] Paula J. Shadle Overview of GMPs, BioPharm International. Nov 15, 2004. http://www.biopharminternational.com/biopharm/artile/articleDetail.jsp?id=134225andpageID=2.

[3] Website: World Health Organisation. http://www.who.int/medicines/areas/quality_safety/quality_assurance/production/en/index.html.

[4] The Code of Federal Regulations Title 21-Food and Drugs Chapter 1 - Food and Drug Administration Department of health and human services Subpart C - Drugs: General part 211 Current Good Manufacturing Practice for Finished Pharmaceuticals Website. http://www.accessdata.fda.gov/scripts/cdrh/cfdocs/cfcfr/CFRSearch.cfm.

[5] EudraLex; The Rules Governing Medicinal Products in the European Union Volume - 4; Good Manufacturing Practices Part I Basic Requirements for Medicinal Products. http://ec.europa.eu/enterprise/sectors/pharmaceuticals/documents/eudralex/vol-4/index_en.htm.

[6] ICH Q7 Good Manufacturing Practice Guide For Active Pharmaceutical Ingredients. Current step 4 version; November 2000 Web site. http://www.ich.org/LOB/media/MEDIA433.pdf.

[7] (2004). Quality assurance of pharmaceuticals. A compendium of guidelines and related materials; ‘Good manufacturing practices and inspection’. Updated edition.

[8] Schedule M Good Manufacturing Practices and Requirements of Premises, Plant and Equipment for Pharmaceutical Products; The Drugs And Cosmetics Act And Rules The Drugs And Cosmetics Act, 1940, (As Amended Up To The 30th June, 2005) And The Drugs And Cosmetics Rules, 1945; (As Amended Up To The 30th June, 2005). http://www.cdsco.nic.in/html/DrugsandCosmeticAct.pdf.

[9] PIC/S Pharmaceutical Inspection Convention Pharmaceutical Inspection Co-operation Scheme “Guide to Good Manufacturing Practice for Medicinal Products PE 009-9 Part-I; September 2009. http://www.picscheme.org/publication.php?id=4.

[10] Guidance for Industry: Manufacturing, Processing or Holding Active Pharmaceutical Ingredient, Draft Guidance; USFDA, Centre for Drug Evaluation and Research CDER March 1998.

[11] White Paper, The 10 Golden Rules of GMP; PharmOut Pty Ltd www.pharmout.com.au Version-01, 2008. www.pharmout.com.au.

[12] Documentation and Records: Website of GMP Online Consultancy. http://www.gmp-online-consultancy.com/gmp/Documentation-Records.htm.

[13] Documentation Requirement; Website of GMP quality. http://www.gmp-quality.com/documentation.html.

